# Designing Participatory Aging Research on Exclusion From Social Relations: A Citizen Science Project

**DOI:** 10.1093/geroni/igab046.1505

**Published:** 2021-12-17

**Authors:** Anna Urbaniak

**Affiliations:** University of Vienna, Wien, Wien, Austria

## Abstract

Many policies and initiatives aim at enhancing the social participation of older adults. Despite this growing interest in increasing social inclusion and combating social exclusion in older age, the voices of socially-excluded older adults and their experiences remain underrepresented in research. Based on data from the Austrian research project “Socially Excluded Older Adults: Voices and Experiences” (SEVEN), I reflect on what it means to co-create research with the hard-to-reach populations of socially excluded older adults. Data discussed is derived By inviting this group to participate in each stage of the research, the project develops an innovative approach that, on the one hand, facilitates and advances ways for socially-excluded older adults to express their voices, thereby empowering them and their self-advocacy, and, on the other hand, creates research insights that are able to grasp the life worlds of older, socially excluded adults more accurately.

